# Virtual embodiment training is associated with relative alpha power modulation

**DOI:** 10.3389/fnhum.2025.1537463

**Published:** 2025-05-19

**Authors:** Soraya Miremadi, Kai Wai Yang, Akshat Kalra, Sri Lasya Malladi, Julia A. Scott

**Affiliations:** ^1^Brain and Memory Care Lab, Department of Neuroscience, Santa Clara University, Santa Clara, CA, United States; ^2^Brain and Memory Care Lab, Department of Electrical and Computer Engineering, Santa Clara University, Santa Clara, CA, United States; ^3^Brain and Memory Care Lab, Department of Computer Science and Engineering, Santa Clara University, Santa Clara, CA, United States; ^4^Brain and Memory Care Lab, Department of Bioengineering, Santa Clara University, Santa Clara, CA, United States

**Keywords:** virtual embodiment, EEG, chronic pain, virtual reality, alpha power band

## Abstract

**Introduction:**

Virtual Reality mediated virtual embodiment training (VR-VET) is designed to reduce chronic pain, yet a neuroimaging marker predictive of outcomes or associated with clinical changes in pain has not been validated. This study considers four candidate EEG metrics that are associated with cognitive states of mental imagery, chronic pain intensity, and stress states. VR-VET with EEG enables measurement of these metrics and collection of kinematic data. Kinematic data serves as an indicator of functional movement. In a healthy population, this study assessed neuroimaging markers for cognitive processes involved in VET or pain perception.

**Methods:**

EEG was collected in 16 healthy individuals during VR-VET. Candidate EEG metrics were computed. Position data for each hand was used to calculate smoothness of movement within each activity. EEG metrics and smoothness were compared between the breathwork activity and activities with active movement of arms.

**Results:**

Relative global alpha was significantly different in all VET activities compared to breathwork (*p* < 0.001). Specifically, relative posterior alpha power (*p* < 0.001) and relative mu (*p* = 0.026) were significantly lower in all active conditions. Smoothness of the active arm varied across VET activities and was reduced compared to breathwork (*p* < 0.001).

**Discussion:**

Neuroimaging markers are feasible to investigate VET mechanisms during movement. Relative global alpha is sensitive to VET states and may be related to motor imagery tasks or visual attention, making it a relevant EEG metric in the study of VET.

## 1 Introduction

On a cognitive level, various processes and corresponding pathways characterize our experience of pain, but chronic pain in particular goes beyond nociception (Apkarian et al., 2012). Several psychological phenomena and behavioral symptoms accompany the clinical status of chronic pain, with perception, cognition, attention, emotion, learning, memory, and motivation affected (Simons et al., 2014). A central aspect of somatic pain perception is mediated by body representation and can be influenced by surrogate and virtual interactions (Beccherle and Scandola, 2024). Non-pharmacological interventions can target a particular psychological process, and neuroimaging methods like electroencephalography (EEG) may potentially detect patterns of brain activity associated with motor imagery and body representation (Pamplona et al., 2022). One such intervention is virtual embodiment training (VET).

VET centers on embodiment, comprising body ownership, self-location, and self-agency, in a virtual environment. When an individual embodies an avatar, meaning they perceive their virtual body to be their own, feel they are located in their virtual body, and feel they have control over their virtual body, various visualizations can act on their perception to change their memory of pain (Kilteni et al., 2012). Early evidence suggests improved pain outcomes in the use of VET for back pain rehabilitation (Bordeleau et al., 2022), as well as improved functional movement in patients with movement-related shoulder pain (De La Campa et al., 2023). More recently, a clinical trial demonstrated improved lower back function and lowered pain intensity after eight sessions of VET (Saby et al., 2024). While VET has been shown to improve pain management in unresolved chronic pain conditions, the mechanisms explaining its efficacy are incompletely studied.

As an intervention for chronic pain, VET acts on pain perception systems, as well as on cognitive states of mental imagery and planning. A clear signature of motor cognitive processes is alpha oscillation desynchronization in sensorimotor regions engaged by VET. The mu rhythm comprises alpha oscillations over the sensorimotor cortices, undergoing event-related desynchronization (i.e., mu suppression) during the observation or execution of movement, as well as in pain perception and empathy (Hobson and Bishop, 2017). In one study, pain-induced suppression of mu was observed, along with a suppression of alpha and low beta oscillations in sensorimotor and visual cortical areas (Ploner et al., 2006). In another study, sensorimotor alpha was suppressed in anticipation of pain, with the extent of suppression predicting the subjective perception of pain intensity (Babiloni et al., 2006). Accordingly, cortical alpha rhythms have been targeted in mindfulness therapy to redirect somatosensory attention away from chronic pain (Kerr et al., 2013). Alpha desynchronization has also been observed over sensorimotor areas during movement preparation in both motor imagery and motor tasks, marking a shift from rest to cognitive planning (Pfurtscheller and Neuper, 1997).

Collectively, previous research implicates the alpha frequency range (8–12 Hz) in both pain perception and mental imagery and planning, prompting our exploration of four alpha measures associated with cognitive states of mental imagery (absolute mu power) (Yin et al., 2016), chronic pain intensity (global relative alpha, absolute frontal alpha) (Jensen et al., 2013), and stress states (frontal alpha asymmetry) (Zhang et al., 2018). Thus, our study considers the fitness of these candidate EEG metrics to investigate cognitive processes engaged in VET or pain perception in a virtual reality application for VET (VR-VET). The program is a collection of activities that actively engage the upper limbs and visualize the limb movement through mirroring of the avatar limb; it also includes mindfulness activities as a coping mechanism, which serves as a non-movement contrast condition. Concurrent EEG and VR touch controller-based kinematic data was used to characterize the neural correlates and behavior associated with the program. The hypothesis that motor imagery and pain-associated brain activity metrics would be modulated in VR-VET was tested in a healthy young adult sample to initially demonstrate the feasibility of the experimental design and analysis prior to investigation in a clinical population.

## 2 Methods

### 2.1 Participants

A total of 25 participants (10 women; 2 left-handed) were recruited from Santa Clara University for the pilot phase of this study (Supplementary Table 1). They were between the ages of 18 to 30, variable in gender, race, and ethnicity, and fluent in English. Participant inclusion criteria included comfort tolerance and proper fit of corrective lenses in VR. Exclusion criteria included a head circumference greater than 58 cm, major neurological disease, traumatic brain/head injury, motor impairments, hair extensions, lack of COVID vaccination, respiratory/eye infection symptoms, lice, and stimulation sickness. All participants initially met the study screening criteria (without chronic pain or mobility impairments) and participated in KVET™ Virtual Embodiment Training. This study was approved by the institutional review board of Santa Clara University (approval number: 21-10-1696). We obtained written informed consent from each participant prior to enrollment.

### 2.2 Procedure

**Figure 1:**
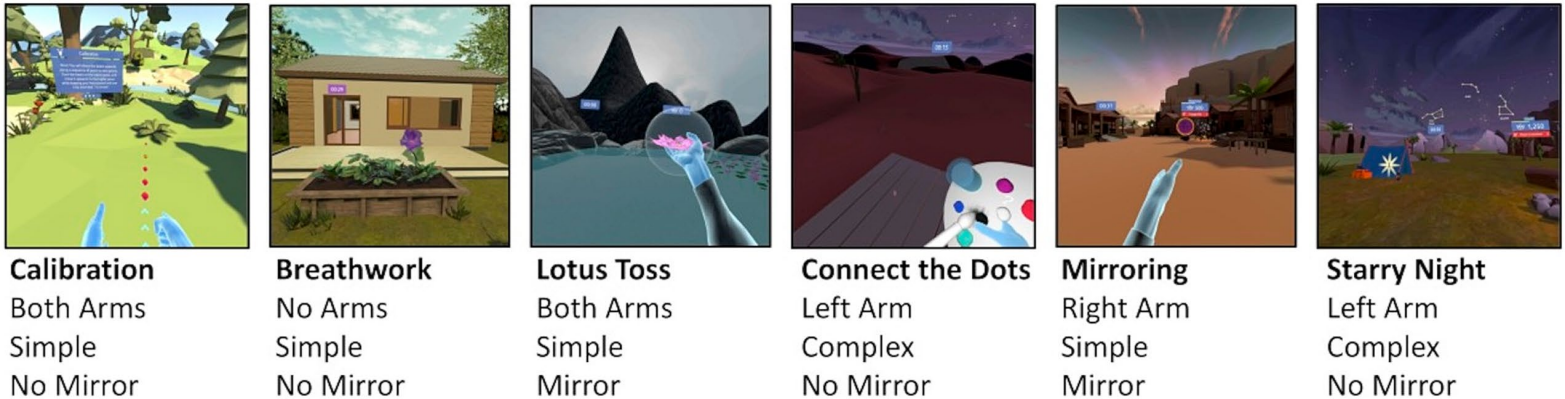
KarunaHOME VET activities. Select activities of the VET program used in this study. Each activity represented a different combination of parameters based on active arm(s), simple or complex movements, and application of mirroring visualization. Each activity listed, except breathing, was done by the participant for one trial of three minutes. Breathing alternated between activities for one-minute trials.

Participants completed two sessions of a KarunaHOME upper extremity coaching program on a Meta Quest 2 virtual reality (VR) headset with touch controllers. The first session was a trial run, followed by a simultaneous VR-EEG experimental session. They performed a series of activities designed to exercise their range of motion and promote neuromotor function. VET activities varied in which arm was used and whether virtual mirroring was applied (Figure 1). Activities included Calibration, Lotus Toss (LT), Connect the Dots (CTD), Mirroring (M), and Starry Night (SN).

Calibration assesses a participant’s effective range of motion and calibrates the following exercises. Participants keep their arm fully extended as they move it through a gem path. Both arms are used to execute simple movements. Lotus Toss presents mirrored movements for cognitive and physical retraining. Participants grab and release lotus flowers ipsilaterally or contralaterally (i.e., mirrored motion). In the activity, right and left arms are used alternately. This is considered a simple movement since it is one straight motion. Connect the Dots recruits multiplanar motion in complex patterns. Participants continuously drag a paintbrush with their nondominant arm through numbered targets to form uniplanar or multiplanar shapes. The arm consequently reaches and twists to execute complex movements. Mirroring involves mirrored movements to decrease resistance in exercising the non-dominant arm. Participants shoot a laser beam through fiery targets using their dominant arm to control the contralateral nondominant virtual arm. This is a simple movement of pointing. Starry Night exercises multiplanar motion similar to Connect the Dots. Participants continuously direct a wand through numbered stars to create multiplanar constellations with their non-dominant arm. These are complex movements in three-dimensional space. The total program length was 20 minutes. Between each upper extremity exercise, participants completed a guided breathing exercise (BAVG) that did not expressly engage the upper extremities. The guided breathing exercise is a meditation activity staggered throughout the sequence to relax the nervous system and mind. Participants inhale as a purple flower opens and exhale as it closes. Arms are not actively engaged and rather rest at the participants’ sides. As the shoulders rise and fall in the breathing cycle, the VR controllers move in concert. This residual movement is what is captured by the position tracking that is input to the smoothness calculation.

Additionally, participants completed a pain sensitivity questionnaire (PSQ) in which they assessed their level of pain in a variety of imagined situations on a 10-point scale (0 = no pain, 10 = worst pain imaginable) (Ruscheweyh et al., 2012; Ruscheweyh et al., 2009).

### 2.3 EEG recordings

EEG was collected from ANT Neuro’s saline-based 24-channel waveguard™ net (Supplementary Figure 1) and eego™ 24 amplifier. Electrodes were positioned in accordance with the International 10/20 System and impedances were kept below 30 kΩ. After a preliminary impedance check, data was recorded and streamed for the length of the training program at a sampling rate of 500 Hz. EEG signals were amplified and digitized.

### 2.4 EEG data processing and analysis

One participant experienced motion sickness and did not complete the sessions, while another had unsuitable impedances due to hair extensions. Moreover, data from two participants did not upload to the Karuna server. Data from five other participants was rejected due to intolerable amplitudes across EEG channels. Thus, with 9 participants excluded from the analysis, 16 participants’ data was analyzed (7 women; one left-handed) (Supplementary Table 1).

**Figure 2:**
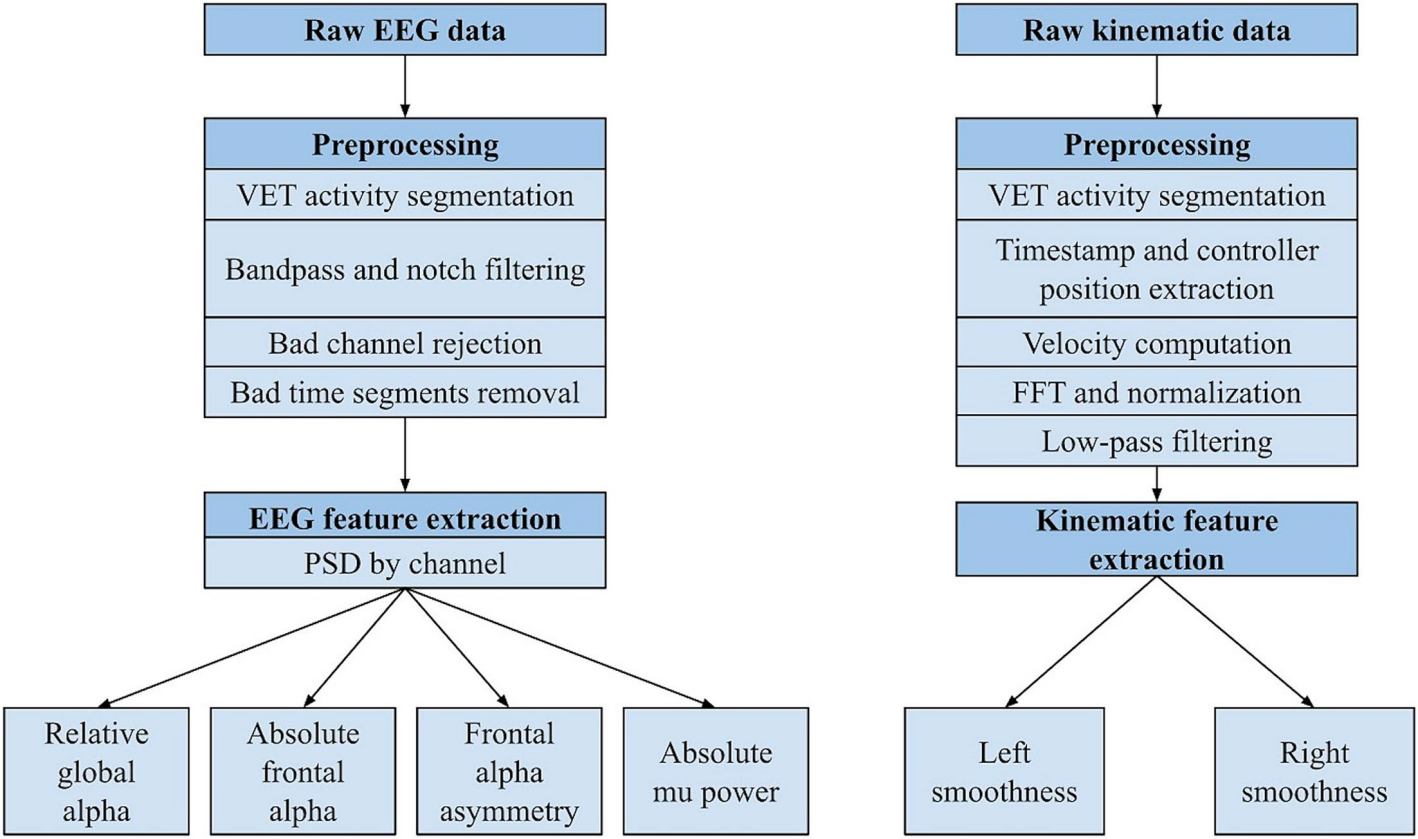
Processing pipeline for EEG and kinematic data. PSD = power spectral density, FFT = fast Fourier transform.

EEG data was processed and analyzed using MNE library tools (Figure 2). Raw EEG data was time-synchronized with kinematic data and processed in MNE-Python. Bandpass (1–70 Hz) and notch filters (60 Hz) were applied to the merged data. Channels with impedances above 30 kΩ were rejected. Additionally, T9 and T10 were rejected across all participants due to consistently poor contact with the head. Next, amplitude-based rejection was applied to the data. For every channel, a second (comprising 500 data points) was marked as bad (i.e., contaminated) if the minimum and/or maximum data point exceeded 3 standard deviations of the mean. Central and frontal channels were prioritized, foundational to the calculation of candidate metrics. If a dataset was contaminated for 30% or more of its total seconds across these 11 channels, then it was excluded from the analysis. Datasets with central and frontal channel data below this threshold were included. Parietal, temporal, and posterior channels were rejected from a dataset if they were contaminated for 40% or more of the total seconds. Commonly bad seconds were dropped across all channels, corresponding to various activity segments (Supplementary Table 2).

Power spectral density analysis followed. Frequency bands were defined as follows: delta (1-4 Hz), theta (4–8 Hz), alpha (8–12 Hz), beta (12–30 Hz), and gamma (30–50 Hz). Candidate EEG metrics were calculated by VET activity using SciPy library functions. Relative global alpha was computed as the ratio of alpha power averaged across all channels to the total power (1–50 Hz) averaged across all channels. Absolute frontal alpha was computed as the average of alpha power across all frontal channels. Frontal alpha asymmetry was computed as the log quotient of alpha power between F8 (right) and F7 (left) channels (Yin et al., 2016). Absolute mu power was computed as the average of alpha power across channels C3 and C4.

In addition to the candidate EEG metrics, all other relative global bandpowers (delta, theta, beta, and gamma) were computed. To assess the contribution of regional alpha rhythms to relative global alpha, relative frontal alpha (Fp1/Fp2), relative mu (C3/C4), and relative posterior alpha (O1/O2) were computed. Regional relative alphas were calculated similarly to relative global alpha but limited to the channels defined for each region.

### 2.5 Kinematic data processing and analysis

Kinematic data was simultaneously recorded from the Meta Quest 2 and uploaded to the Karuna server. The processing pipeline was built with pandas and math python libraries (Figure 2). VET activities were segmented. Time and VR touch controller positions were extracted to compute velocity. For each activity and each VR controller (right and left), velocity was calculated by applying the root mean square of xyz positions from each moment (72 Hz). Any instances of NaN and infinity values were removed from the movement data. Then, a fast Fourier transform magnitude spectrum of the data was computed (*nfft* function), followed by normalization. Low-pass filtering (10 Hz) and amplitude threshold (0.05) was applied to remove high-frequency noise. Finally, smoothness of both right and left arm movements was calculated for each activity using the spectral arc length method, which is calculated by the Euclidean distance between each pair of consecutive points in the frequency-magnitude space. The final metric is a negative value, such that more negative values represent greater smoothness (Balasubramanian et al., 2015).

### 2.6 Statistical analysis

Differences between breathwork and VET activities were tested through multivariate GLM for EEG metrics, regional relative alphas, relative global bandpowers, and smoothness. All variables, except frontal alpha asymmetry, were log-transformed due to high skewness and kurtosis in the original data. Each model tested for the main effects of age, sex, and activity. A bivariate correlative analysis between PSQ and EEG metrics for breathwork was conducted with a nonparametric Spearman rho coefficient because the PSQ scores were not normally distributed. No significant association was found between PSQ and the EEG metrics for breathwork (Supplementary Table 6).

## 3 Results

EEG metrics and Smoothness metrics by activity are reported in Supplementary Table 3.

**Figure 3:**
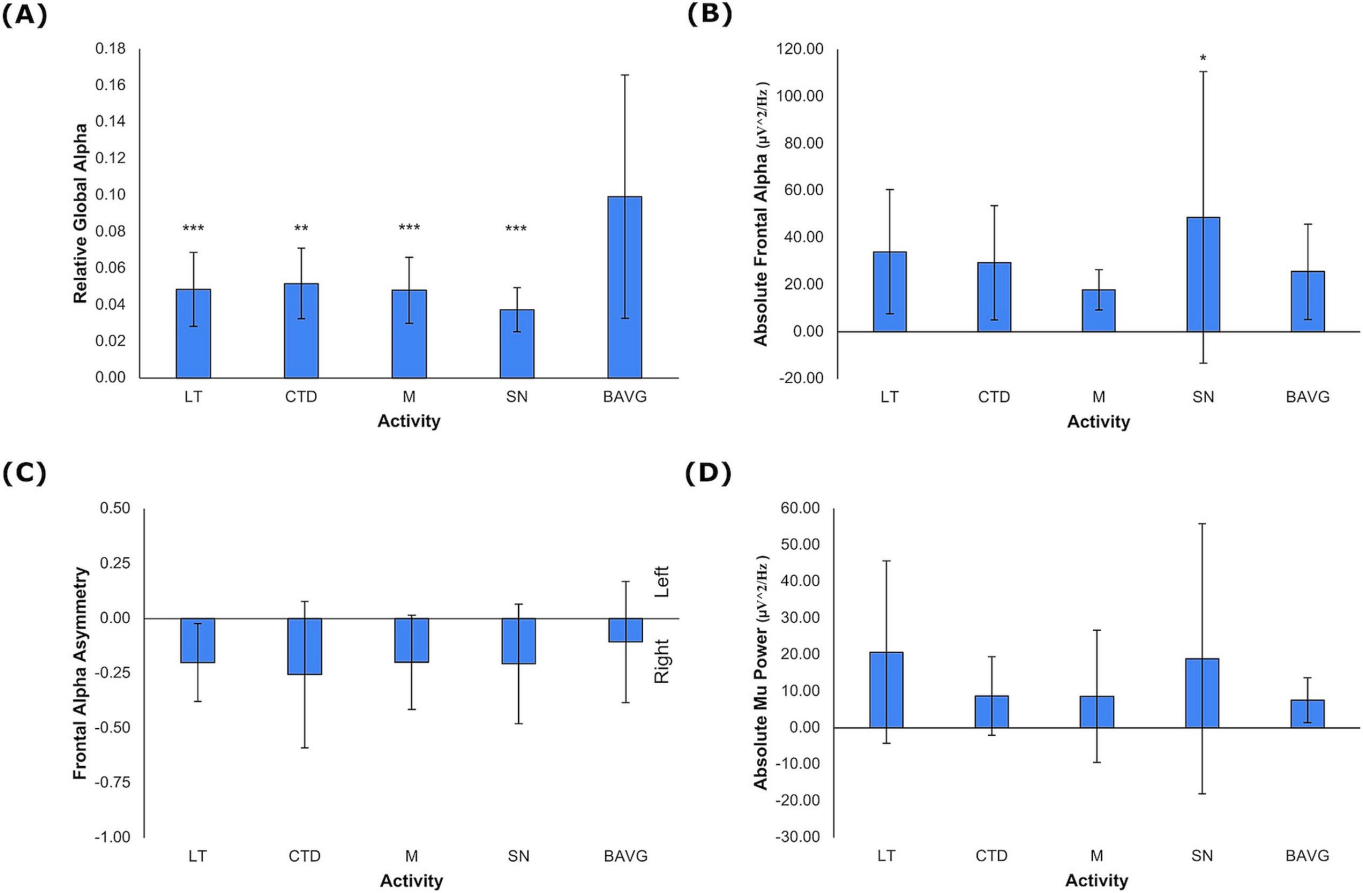
EEG Metrics by VET Activity. **(A)** Relative global alpha, **(B)** absolute frontal alpha, and **(C)** frontal alpha asymmetry, and **(D)** absolute mu power. Negative values for frontal alpha asymmetry denote rightward activation (mean +/− 1 stdev). Statistics performed by mixed GLM, comparing breathwork to active conditions (*p < 0.05, **p < 0.01, ***p < 0.001). LT = Lotus Toss, CTD = Connect the Dots, M = Mirroring, SN = Starry Night, BAVG = Breathwork.

The model for the main effect of activity, controlling for sex and age, was significant in the multivariate general linear model for candidate EEG metrics (*F* = 3.206, η^2^ = 0.151, *p* < 0.001). Significant between-subjects effects of VET activity emerged for relative global alpha (*F* = 7.777, η^2^ = 0.299, *p* < 0.001), absolute frontal alpha (*F* = 3.145, η^2^ = 0.147, *p* = 0.019), and absolute mu power (*F* = 2.538, η^2^ = 0.122, *p* = 0.047). There was not a significant effect of VET activity for frontal alpha asymmetry. Only relative global alpha was lower in VET activities compared to breathwork, indicating alpha power association with VET (Figure 3A, LT, M, SN *p* < 0.001, CTD *p* = 0.001). Absolute mu and frontal alpha were singularly lower during mirroring as compared to breathwork (Figures 3B, *p* = 0.002, Figure 3D, *p* = 0.034). Additionally, relative global alpha (*F* = 14.981, η^2^ = 0.170, *p* < 0.001) and absolute frontal alpha (*F* = 29.205, η^2^ = 0.286, *p* < 0.001) displayed significant intersubject variability (Figure 3C).

The next model evaluated regional relative alpha aggregated over select channels as described in Methods. Activity (*F* = 3.528, η^2^ = 0.174, *p* < 0.001), sex (*F* = 3.235, η^2^ = 0.128, *p* = 0.028), and age (*F* = 6.145, η^2^ = 0.226, *p* < 0.001) showed significant main effects. All VET activities compared to breathwork were significantly lower for both relative mu (*F* = 3.963, η^2^ = 0.189, *p* = 0.006) and relative posterior alpha (*F* = 8.530, η^2^ = 0.334, *p* < 0.001) (Supplementary Table 4). Relative frontal alpha was not associated with activity. Additionally, relative mu decreased slightly with age (β = −0.040, η^2^ = 0.070, *p* = 0.026).

In a separate model, all frequency bands (delta, theta, alpha, beta, gamma) were examined to better understand the specificity of the alpha band. A significant effect of activity (*F* = 3.247, η^2^ = 0.187, *p* < 0.001) was reported for relative global delta (*F* = 3.136, η^2^ = 0.147, *p* = 0.019), relative global alpha (*F* = 7.777, η^2^ = 0.299, *p* < 0.001), relative global beta (*F* = 4.392, η^2^ = 0.213, *p* = 0.001), and relative global gamma (*F* = 2.849, η^2^ = 0.135, *p* = 0.030). Relative global theta was not significantly associated with activity. Only relative global alpha was significantly lower in all VET activities compared to breathwork (*p* < 0.001) (Supplementary Table 5). Age was significantly associated with the EEG metrics (*F* = 12.555, η^2^ = 0.476, *p* < 0.001). Delta (β = 0.015, η^2^ = 0.145, *p* < 0.001) and theta (β = 0.025, η^2^ = 0.075, *p* = 0.010) frequency bands (1–8 Hz) were positively associated with age, while beta (β = −0.056, η^2^ = 0.288, *p* < 0.001) and gamma (β = −0.083, η^2^ = 0.385, *p* < 0.001) frequency bands (12–50 Hz) were negatively associated with age. A main effect of sex was also significant (*F* = 11.842, η^2^ = 0.462, *p* < 0.001). The female group was associated with higher relative power in delta (β = 0.083, η^2^ = 0.173, *p* < 0.001) and theta (β = 0.114, η^2^ = 0.075, *p* = 0.017) frequency bands and lower relative power in beta (β = −0.230, η^2^ = 0.220, *p* < 0.001) and gamma (β = −0.266, η^2^ = 0.385, *p* < 0.001) bands. The relative global alpha had no significant association with age or sex.

**Figure 4:**
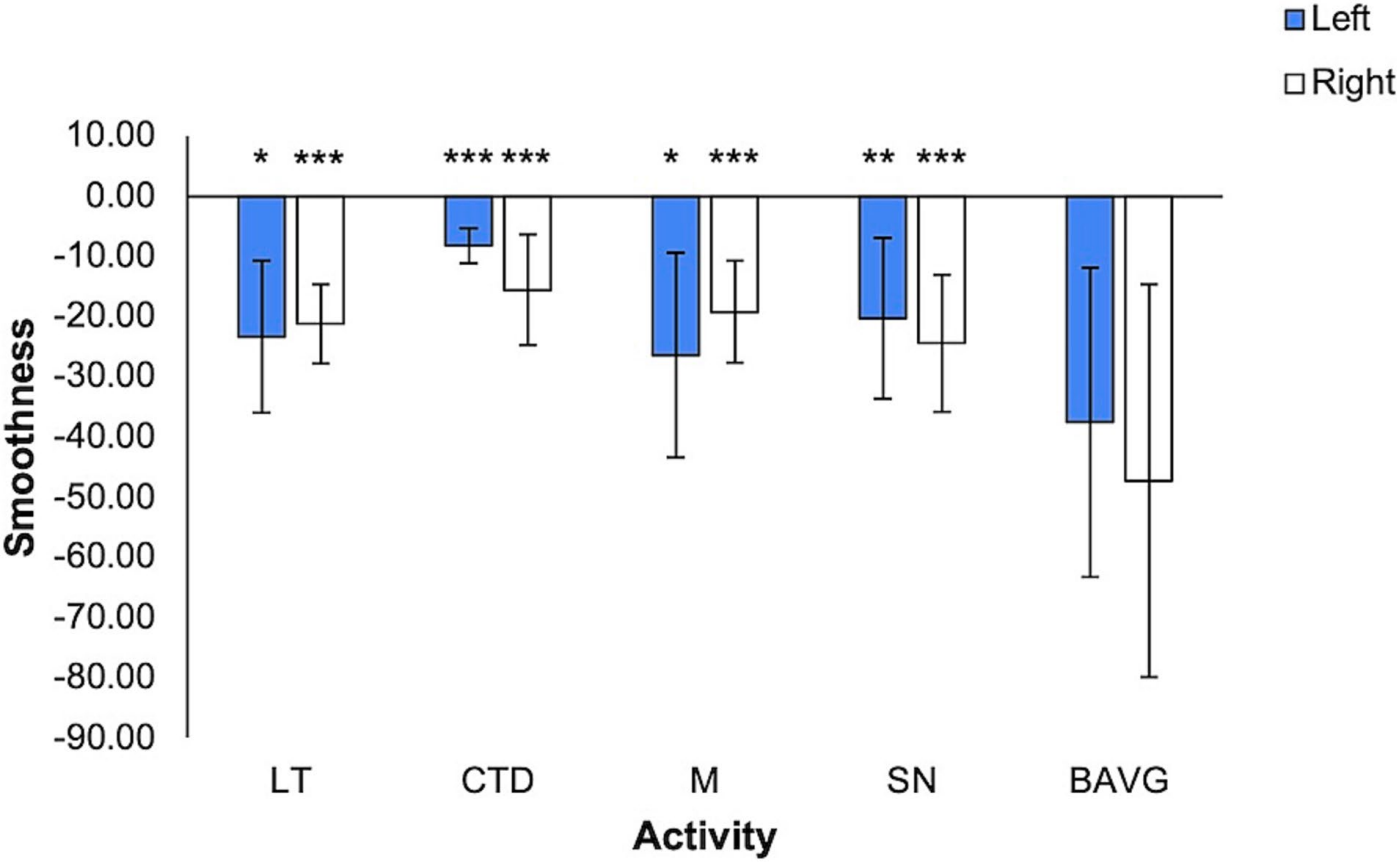
Smoothness by VET Activity. More negative values denote greater smoothness (mean +/− 1 stdev). Statistics performed by mixed GLM, comparing breathwork to active conditions (*p < 0.05, **p < 0.01, ***p < 0.001). LT = Lotus Toss, CTD = Connect the Dots, M = Mirroring, SN = Starry Night, BAVG = Breathwork.

Smoothness ranged on average from −8.22 +/− 2.97 to −47.25 +/− 32.66 (Figure 4). In the model (right hand: *F* = 9.040, η^2^ = 0.462, *p* < 0.001; left hand: *F* = 10.660, η^2^ = 0.467, *p* < 0.001), smoothness was significantly associated with activity (right hand: *F* = 12.577, η^2^ = 0.408, *p* < 0.001; left hand: *F* = 15.963, η^2^ = 0.467, *p* < 0.001). Right-hand smoothness was lower in all VET activities compared to breathwork (*p* < 0.001). Left-hand smoothness difference compared to breathwork varied by activity (CTD *p* < 0.001, LT *p* = 0.024, M *p* = 0.031, SN *p* = 0.002).

Pain sensitivity, as indexed by PSQ score, ranged from 2.72 to 5.93 on a 10-point scale (Supplementary Table 6), accurately characterizing the healthy study population.

## 4 Discussion

This study demonstrated the feasibility of four neuroimaging markers to investigate VET mechanisms under the challenge of free movement in VR. Relative global alpha was sufficiently sensitive to differentiate VET state from non-embodied visualization (Figure 3A). The global measure was driven primarily by posterior alpha and secondarily by mu (alpha in the sensorimotor region). These findings indicate that avatar visualization during motor planning is associated with mu desynchronization in contrast to a non-motor visually guided task. While metrics associated with stress (frontal asymmetry) or pain sensitivity (absolute frontal alpha) were not linked to VET in this experiment with healthy, young adults, the metrics were accurately measured under these conditions and may be included in future studies with a study group of chronic pain patients.

### 4.1 Motor imagery as a biomarker

The thesis of virtual embodiment training for chronic pain management is that synchronizing a user’s movement with adaptive representations of the movement (such as a digital avatar) will change the perception of one’s ability to do the movement. The repetitive visual stimuli of non-painful synchronized movement effectively produce new sensorimotor memories as was demonstrated in early mirror box experiments for phantom limb pain (Ramachandran and Rogers-Ramachandran, 1996). Pain is mediated by numerous cognitive processes of which few are modifiable by behavioral interventions (Apkarian et al., 2012). The memory of pain is one of those targeted dimensions.

Thus, effectively engaging motor imagery leads to decreased pain perception (Saby et al., 2024; Matamala-Gomez et al., 2019; Vassantachart et al., 2022; Trujillo et al., 2020). Since motor imagery and planning have a unique and robust neuroimaging signature in mu desynchronization, it is a candidate biomarker for intervention mechanism or as a prediction of responsiveness to VET. In this foundational study of VET in a healthy population, global alpha desynchronization differentiated unambiguously between tasks that engaged movement of limbs with an avatar representation and embodied activities that lacked these two characteristics. It did not differentiate between tasks with and without mirroring (contralateral representation of avatar limb). This suggests that global alpha may serve as a general measure of motor imagery. Yet, sensorimotor and posterior alpha may be more precise biomarkers that measure user engagement with VET.

Relative global alpha effect was seen in relative mu and posterior alpha rhythms, but the frontal alpha rhythm did not display the same sensitivity to movement. Sensorimotor (mu) and visual (posterior) systems were engaged in embodiment, as individuals visualized stimuli and their own limbs in the virtual environment while exercising sensorimotor control of the avatar. Motor imagery and planning of the upper limb would be most closely oriented to C3/4. Consistent mu desynchronization in VET underscores the active exercise of motor imagery, which may be guided by visually tracking the movement of the avatar. This effect in alpha is associated with goal-directed tasks and learning more broadly (Yin et al., 2016), which suggests that the VET is effectively forming new somatic memories of the affected limb. In a population with chronic pain, a reasonable hypothesis to test is that somatic memories formed during VET reduce pain perception (Simons et al., 2014; Beccherle and Scandola, 2024; Mayaud et al., 2019).

Higher amplitudes of posterior alpha manifest as the default mode rhythm of the awake brain during eyes-closed, resting conditions (Garcia-Rill, 2015). When eyes are open, posterior alpha desynchronizes. Posterior alpha power was lower during VET activities than during breathwork, which lacked any body representations in virtual avatars. This effect does not exclude general task-related modulations of alpha. This suggests that the VET conditions required greater spatial attentional engagement compared to breathwork, likely based on the complexity of the task (Jensen, 2024). In other words, alpha desynchronization during activity reflected a shift in visual attention to active tasks and the corresponding allocation of mental resources to effectively respond to the embodied stimuli.

### 4.2 Limitations

A key limitation of our study was data loss due to motion, introducing motion-related noise and artifacts. To maintain the integrity of data, rigorous thresholds were applied to EEG signals, leading to the rejection of hundreds of seconds of data. Further, with active conditions routinely occurring in 3-min blocks and breathing segments in 1-min blocks, candidate metrics were not time-locked, but averaged over long periods. Thus, specific events within the activities could not be differentiated in analysis. Another limitation was the healthy population assessed, lacking a comparative pain population. Pain sensitivity characterization was limited to the PSQ, which relies on self-assessment of pain perception that is remembered or imagined. At this point, in the absence of data from chronic pain patients, only hypotheses can be derived regarding the relevance of these metrics to chronic pain.

### 4.3 Future directions

VR-VET is a unique and valuable non-pharmacological intervention, leveraging embodied visualizations and movements to encourage physical exercise and effectively modify pain perception (Adamovich et al., 2009; Rodriguez et al., 2023). While motion is an inherent element of embodiment, it impacts the quality of EEG data. However, future studies may implement an event-related analysis that rejects short epochs of data and preserves many clean and time-locked segments. Therefore, within each embodiment activity, tailored in complexity and laterality of motion, individual tasks can be analyzed to further elucidate mechanisms of VET.

In addition to the four candidate metrics proposed here, peak alpha frequency (PAF) would be a likely trait predictor of pain sensitivity. Future studies with an eyes-closed condition would be suitable to measure PAF, which is inversely related to pain sensitivity (Furman et al., 2018; McLain et al., 2022).

Next, steps will be to conduct a study with a clinical population of upper limb chronic pain to understand whether these metrics are modulated by or predictive of responses to VR-VET. A variety of possible activities demonstrate the versatility and therapeutic power of VET to promote and restore functional movement of non-dominant, injured, or phantom limbs (Bordeleau et al., 2022; Vassantachart et al., 2022; Chau et al., 2020). Moreover, smoothness—accurately calculated and sensitive to a range of 3D motions—can potentially serve as a measure of functional recovery and a useful adjunct to range of motion metrics by capturing hesitations and inaccuracies to perform avoidant or protective movements (De La Campa et al., 2023; Saby et al., 2024). Potentially, EEG-based biomarkers may be strongly correlated with kinematic measures of functionality.

### 4.4 Conclusion

The four neuroimaging markers of pain perception under investigation (relative global alpha, absolute mu power, absolute frontal alpha, and frontal alpha asymmetry) were robustly measured during movement, with relative global alpha in particular sensitive to VR-VET states. Concurrent EEG recordings enabled the exploration of these markers as neural correlates of VET, demonstrating that relative mu and relative posterior alpha power were selectively suppressed during motor imagery. This finding provides evidence that motor imagery may be a mechanism to alter perception by mediating new somatic memories of movement.

## Data Availability

The raw data supporting the conclusions of this article will be made available by the authors, without undue reservation.
