# Supplementary material for: Virtual embodiment training is associated with relative alpha power modulation
Source: Front Hum Neurosci. 2025 May 19;19:1537463. doi: 10.3389/fnhum.2025.1537463 (PMC12127390; doi:10.3389/fnhum.2025.1537463)
Supplement: Supplementary file 1 [file Data_Sheet_1.DOCX]

## ***Supplementary Material***

*
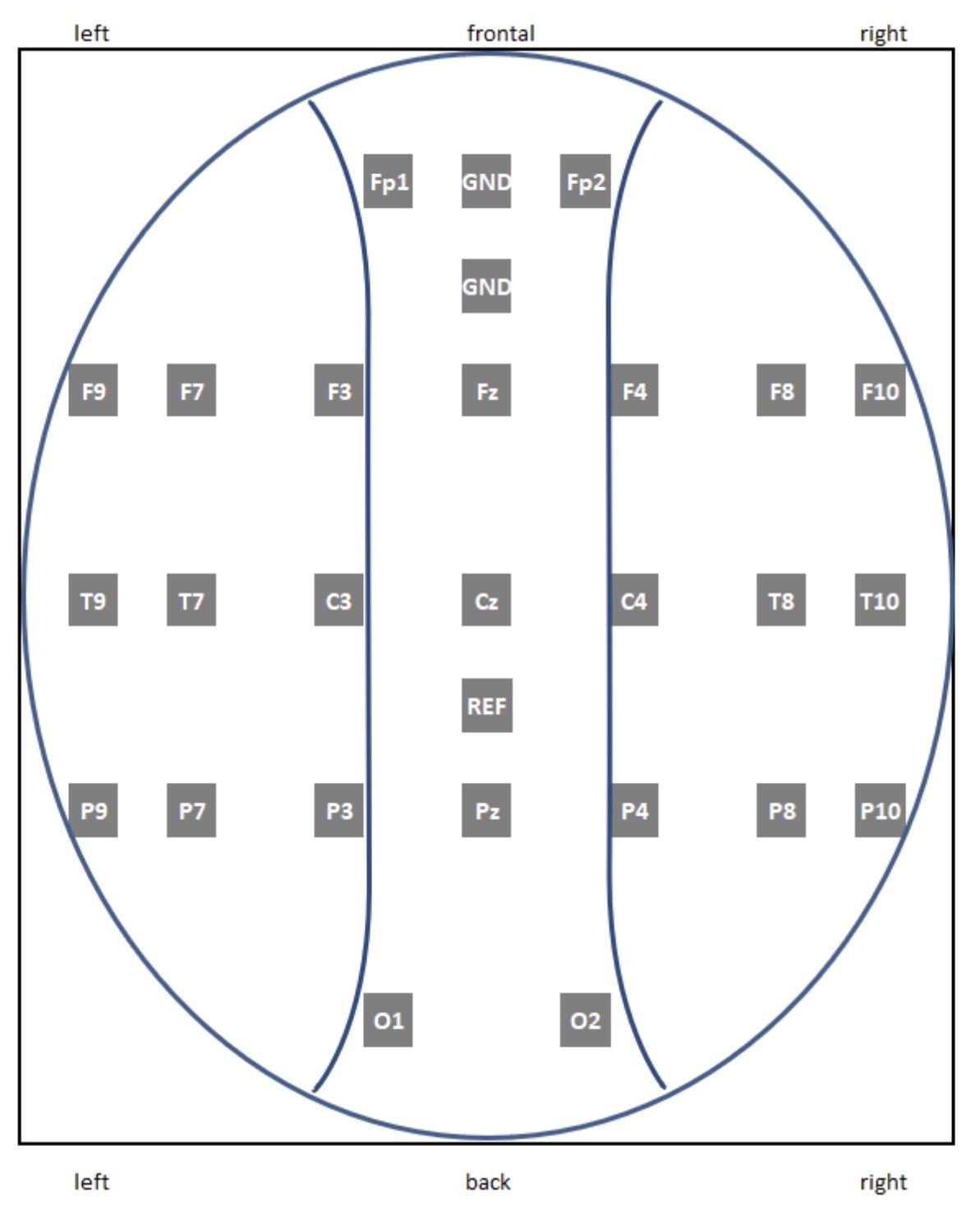
*

**Supplementary Figure. 1:** EEG electrode placement. The ANT Neuro Waveguard net montage with 24 channels used for recording*.*

**Supplementary Table 1.** Sample Demographics.

| **Age (mean (stdev))** | 22.4 (2.5) |
| --- | --- |
| **Sex (N)**  Male  Female  Non-binary | 9  7  0 |
| **Race (N)**  White  Black or African American  American Indian or Alaska Native  Asian  Pacific Islander  Hispanic (Non-White)  Multiethnic  Decline to state  Other | 4  1  0  10  0  0  1  0  0 |
| **Handedness (N)**  Right  Left | 15  1 |
| **Pain sensitivity (mean +/- SD)*** | 4.43 (1.43) |

^*^N=13

**Supplementary Table 2.** Rejection Rate by VET Activity

| **Activity** | **Rejection Rate (%) (mean (stdev))** |
| --- | --- |
| Calibration | 20.67 (17.67) |
| Breathing 1 | 7.38 (7.15) |
| Lotus Toss | 15.04 (16.84) |
| Breathing 2 | 7.93 (8.77) |
| Connect the Dots | 9.76 (12.36) |
| Breathing 3 | 7.11 (9.70) |
| Mirroring | 6.37 (7.60) |
| Breathing 4 | 7.09 (6.86) |
| Starry Night | 13.87 (15.13) |
| Breathing 5 | 6.27 (6.41) |

**Supplementary Table 3.** EEG Metrics and Smoothness by VET Activity (mean (stdev)).

|  | **Activity** | | | | |
| --- | --- | --- | --- | --- | --- |
|  | **Avg Breathing** | **Lotus Toss** | **Connect the Dots** | **Mirroring** | **Starry Night** |
| **Relative global alpha** | 0.10 (0.07) | 0.05 (0.02) | 0.05 (0.02) | 0.05 (0.02) | 0.04 (0.01) |
| **Absolute frontal alpha (10^-12^)** | 25.27 (20.29) | 34.03 (26.38) | 29.38 (24.28) | 17.84 (8.55) | 48.62 (62.00) |
| **Frontal alpha asymmetry (F7/8)** | -0.11 (0.28) | -0.20 (0.18) | -0.26 (0.33) | -0.20 (0.21) | -0.21 (0.27) |
| **Absolute mu power (10^-12^)** | 7.58 (6.16) | 20.67 (24.94) | 8.75 (10.77) | 8.61 (18.11) | 18.89 (36.95) |
| **Smoothness Right** | -47.25 (32.66) | -21.20 (6.56) | -15.57 (9.21) | -19.20 (8.48) | -24.37 (11.39) |
| **Smoothness Left** | -37.54 (25.71) | -23.37 (12.62) | -8.22 (2.97) | -26.39 (17.05) | -20.26 (13.35) |

**Supplementary Table 4.** Relative Global Alpha Sub-Rhythms (mean (stdev))

|  | **Activity** | | | | |
| --- | --- | --- | --- | --- | --- |
|  | **Avg Breathing** | **Lotus Toss** | **Connect the Dots** | **Mirroring** | **Starry Night** |
| **Relative frontal alpha** | 0.02 (0.02) | 0.02 (0.01) | 0.02 (0.01) | 0.02 (0.01) | 0.02 (0.01) |
| **Relative mu** | 0.10 (0.07) | 0.05 (0.03) | 0.06 (0.02) | 0.05 (0.03) | 0.04 (0.02) |
| **Relative posterior alpha** | 0.19 (0.12) | 0.08 (0.03) | 0.08 (0.03) | 0.08 (0.03) | 0.06 (0.03) |

**Supplementary Table 5.** Relative Global Bandpowers (mean (stdev))

|  | **Activity** | | | | |
| --- | --- | --- | --- | --- | --- |
|  | **Avg Breathing** | **Lotus Toss** | **Connect the Dots** | **Mirroring** | **Starry Night** |
| **Relative global delta** | 0.61 (0.09) | 0.65 (0.09) | 0.62 (0.10) | 0.66 (0.10) | 0.71 (0.09) |
| **Relative global theta** | 0.10 (0.02) | 0.13 (0.05) | 0.14 (0.06) | 0.11 (0.03) | 0.12 (0.05) |
| **Relative global beta** | 0.12 (0.04) | 0.10 (0.05) | 0.11 (0.04) | 0.10 (0.04) | 0.07 (0.03) |
| **Relative global gamma** | 0.07 (0.03) | 0.07 (0.04) | 0.08 (0.03) | 0.08 (0.05) | 0.06 (0.04) |

**Supplementary Table 6.** Pain Sensitivity Questionnaire (PSQ) Correlation with EEG Metrics

|  | **PSQ** |
| --- | --- |
|  | Spearman’s rank rho correlation coefficient |
| **Relative global alpha** | 0.10 |
| **Absolute frontal alpha** | -0.25 |
| **Frontal alpha asymmetry** | -0.22 |
| **Absolute mu power** | 0.47 |
